
JOURNAL INFORMATION
==============================
NLM Title Abbreviation: ScientificWorldJournal
Journal ID: ScientificWorldJournal
NLM Journal ID: 101131163
Journal ID: TSWJ

The Scientific World Journal

ISSN: 2356-6140
EISSN: 1537-744X
Publisher: Wiley

ARTICLE INFORMATION
==============================
PMCID: PMC6084244
PMID: 30147612
DOI: 10.1100/tsw.2001.231
Article ID: 695392
Article version: 1
Subjects: Short Report

Glutathione Biosynthesis Influences Replicative Longevity in Saccharomyces Cerevisiae

Zandycke Sylvie M. Van
Smart Katherine A.
School of Biological and Molecular Sciences, Oxford Brookes University, Gipsy Lane Campus, Headington, Oxford OX3 0BP, UK
Publication date: 2001
Electronic publication date: 2001 Oct 17
Volume: 1
First page: 133
Last page: 133
Copyright: Copyright © 2001 Sylvie M. Van Zandycke and Katherine A. Smart.
Copyright year: 2001
License: This is an open access article distributed under the Creative Commons Attribution License, which permits unrestricted use, distribution, and reproduction in any medium, provided the original work is properly cited.
License URL: https://creativecommons.org/licenses/by/3.0/

==============================
Miami *Nature Biotechnology* Short Reports
*TheScientificWorld* (2001) 1 (S3), 127SR
ISSN 1532-2246; DOI 10.1100/tsw.2001.231

### GLUTATHIONE BIOSYNTHESIS INFLUENCES REPLICATIVE LONGEVITY IN *SACCHAROMYCES CEREVISIAE*

Sylvie M. Van Zandycke and Katherine A. Smart

School of Biological and Molecular Sciences, Oxford Brookes University, Gipsy Lane Campus, Headington, Oxford OX3 0BP, UK. Tel: 0044 1865 483248 Fax: 0044 1865 484410
kasmart@brookes.ac.uk

**INTRODUCTION.** The yeast *Saccharomyces cerevisiae* possesses a finite lifespan; the metric of which is the number of times the cell divides and not its chronological age (1). The free radical theory of ageing postulates that reactive oxygen species are causal factors in ageing (2). These species are capable of damaging DNA, protein and lipids within the cell. For defence against these prooxidants, cells contain antioxidant molecule such as glutathione (GSH).

**METHOD.** Mean and maximum lifespans were determined by micromanipulation using the method described by Barker *et al* (3). Total glutathione (2GSH + GSSG) levels were measured using a glutathione assay kit from Calbiochem (Cat no 354102) and were expressed as nmol per cell dry weight. Isolation of age-synchronised cell populations was achieved by rate-zonal sedimentation according to a modified version of Egilmez *et al* (4).

**RESULTS.** The role of GSH on the replicative capacity of *Saccharomyces cerevisiae* has been investigated. A mutant lacking Gshp1 activity, and therefore exhibiting impaired GSH biosynthesis, exhibited a reduced lifespan. A deletion mutant for *GSH2*, which also exhibited impaired GSH biosynthesis, displayed longevity potential similar to that observed for the corresponding wild type. Glutathione reductase activity, which maintains cellular levels of reduced GSH, is not required to assure longevity potential. Indeed, the absence of this enzyme leads to a lifespan extension.

Levels of GSH were measured in young and old *Saccharomyces cerevisiae* cells. An age-related decrease in GSH was demonstrated in age-homogeneous populations of virgin, $2^{nd}$, $4^{th}$ and $6^{th}$ generation old cells.

**DISCUSSION.** Several studies have demonstrated the importance of GSH in the ageing of mammalian cells (5). Our studies revealed that glutathione biosynthesis might influence replicative longevity in *Saccharomyces cerevisiae*. It was observed that *GSH1* but not *GSH2* was essential to assure full replicative longevity. It is postulated that that the accumulation of the GSH precursor γ-glutamylcysteine in the *gsh2* mutant enabled the maintenance of longevity potential in this strain. It is suggested that the improvement of longevity observed with the mutant lacking the *GLR1* gene, involved in GSH recycling, may be due to an increase in total GSH. An age-related decrease in GSH levels occurred throughout replicative lifespan. A similar decrease has previously been observed in many organisms (6) and further work is necessary to understand the mechanisms underlying this phenomenon.

**ACKNOWLEDGEMENT.** S. Van Zandycke was supported by an Oxford Brookes University studentship.

**REFERENCES.**

1. Müller, I., Zimmermann, M., Becker, D., and Flomer, M. (1980) Mech. Ageing Dev. 12, 47-52
2. Harman, D. (1956) J. Gerontol. 114, 298-300
3. Barker, M.G., Brimage, L.J.E., and Smart, K.A. (1999) FEMS Microbiol. Lett. 177, 199-204
4. Egilmez, N.K., Chen, J.B., and Jazwinski, S.M. (1990) J. Gerontol. 45, B9-B17
5. Garcia de La Asuncion, J.G., Millan, A., Pla, R., Bruseghini, L., Esteras, A., Pallardo, F., Sastre, J., and Viña, J. (1996) FASEB J. 10, 333-338
6. Liu, R. and Choi, J. (2000) Free Radic. Biol. Med. 28, 566-574
